# Palliative Care - Need of Awareness in General Population

**DOI:** 10.4103/0973-1075.58465

**Published:** 2009

**Authors:** Harshal T Pandve, Kevin Fernandez, Parvinder Singh Chawla, Samir A Singru

**Affiliations:** Department of Community Medicine, Smt. Kashibai Navale Medical College, Narhe, Pune - 411 041, India

Sir,

According to World Health Organization (WHO), palliative care is an approach that improves the quality of life of patients and their families facing the problem associated with life-threatening illness, through the prevention and relief of suffering by means of early identification and impeccable assessment and treatment of pain and other problems—physical, psychosocial, and spiritual. Palliative care provides relief from pain and other distressing symptoms, affirms life, and regards dying as a normal process. It intends neither to hasten nor postpone death. Rather it integrates the psychological and spiritual aspects of patient care, offers a support system to help patients live as actively as possible until death, and offers a support system to help the family cope during the patients illness and in their own bereavement, by using a team approach to address the needs of patients and their families, including bereavement counseling.[1]

It is a worldwide trend that the majority of cancer patients are in advanced stages of the condition when first seen by a medical professional. For them, the only realistic treatment option is pain relief and palliative care. Effective approaches to palliative care are available to improve the quality of life for cancer patients.[2] To create awareness, World Hospice and Palliative Care Day is celebrated on second Saturday of October every year. The theme for World Hospice and Palliative Care Day, 10 October 2009, is “Discovering your voice.” This Day is for people to engage in events and activities to raise awareness and funds to support the development of hospice and palliative care. All around the world, there are people who need hospice and palliative care, and those who cannot access it. This year, the voices of people living with life-limiting illness, their caregivers, and families will be heard to show the importance of hospice and palliative care and what it is important to them. Policy makers and key people who can influence the development of hospice and palliative care will be encouraged to speak out about this important issue.[3]

Palliative care is the active total care of patients in advanced and incurable stages of cancer. More than 70% of all cancer patients in India require palliative care for relief of pain, other symptoms, and psychosocial distress. The need for education and training in palliative care has been emphasized by the WHO.[4] The concept itself is relatively new to India. We do not have comprehensive health coverage in India. The existing heathcare facilities are more attuned to caring for acute health problems and they play only a limited role in the care of the chronically ill in the society. Those who need continued supportive care spend their lives not in the hospital but in the community among their family and neighbors. Hence the community has a major role in the care of these individuals.[5]

A pilot survey was conducted to determine awareness regarding palliative care among the cancer patients and their relative who had visited one of the tertiary care teaching hospitals in Pune, Maharashtra. The patients or their relatives were asked regarding their awareness about palliative care and availability of palliative care centers. Around 47 patients or their relatives participated in the survey. All the 47 (100%) responded that they were not aware about the palliative care, to whom it is required. Similarly all those responded never heard about availability of any centers providing palliative care.

As rightly stated by Koshi, palliative care as a specialty fondly referred to as “low tech and high touch,” depends on caring compassionate hands and we have many! Winds of change are sweeping albeit silently, and palliative care awareness and delivery is a matter of time before it reaches the farthest corner of India.[6] There are quite a few palliative care centers functioning in India; for example, Trivandrum Institute of Palliative Sciences, Trivandrum, Kerala, runs a clinical service developing and operating community-oriented palliative care services including service of local volunteers and home visits and educational programs in pain and palliative medicine. Karunashraya Palliative care center, Bangalore, Guwahati Pain and Palliative Care Clinic/Society, Guwahati, Cipla Foundation's Cancer Palliative Care Centre, and Hamied Institute, Pune are few more examples of such centers.[7–9] Apart from these centers and institutes, some more are also functioning in different parts of the country.[7]

To use facilities of palliative care to the fullest it is essential to create awareness in general population.
